# MicroRNA152-3p Protects Against Ischemia/Reperfusion-Induced Bbb Destruction Possibly Targeting the MAP3K2/JNK/c-Jun Pathway

**DOI:** 10.1007/s11064-022-03828-1

**Published:** 2022-11-29

**Authors:** Fei Li, Fangfang Zhou, Binbin Yang

**Affiliations:** 1 Department of Neurology, Changzheng Hospital, Naval Medical University, Shanghai, China; 2 Department of Neurology, 2nd Xiangya Hospital, Central South University, Changsha, Hunan China

**Keywords:** MicroRNA152-3p, Cerebral Ischemia/Reperfusion, Blood-Brain Barrier (BBB), Apoptosis

## Abstract

In the current study, we reported that overexpression of miR-152-3p effectively ameliorated neurological deficits and protected blood-brain barrier(BBB) integrity in middle cerebral artery occlusion (MCAO) rats. In an in vitro model, the level of miR-152-3p was significantly decreased in bEnd.3 cells after oxygen–glucose deprivation/reperfusion (OGD/R) insult. miR-152-3p overexpressing bEnd.3 cell monolayers were protected from OGD/R-induced microvascular hyperpermeability. The miR-152-3p-mediated protective effect was associated with lower apoptosis of endothelia by negatively modulating the MAP3K2/JNK/c-Jun pathway.

## Introduction

Cerebral ischemia is a major cause of morbidity and longlasting disability worldwide [1]. Unfortunately, therapeutic options for ischemic stroke are extremely limited [2]. Reperfusion therapies have been the mainstay in eligible acute ischemic stroke patients. Rapid reperfusion, however, leads to deleterious effect on brain function, and produced so-called “reperfusion injury”[3]. The BBB is a multicellular microvasculature that prevents harmful substances from entering the CNS, and plays a pivotal role in maintaining central nervous system homeostasis[4]. Accumulating evidence suggests that BBB disruption is one of the hallmarks of cerebral ischemia/reperfusion (I/R), which exacerbates brain injury and leads to subsequent neurological impairment [5, 6]. Brain microvascular endothelial cells (BMECs) held together by tight junctions (TJs) are the scaffold of the BBB [7]. BMEC survival is closely relevant to BBB homeostasis[8, 9]. Hence, attenuating BMEC apoptosis is beneficial for stabilizing the BBB after cerebral ischemia.

MicroRNAs (miRNAs), a novel class of noncoding RNAs of approximately 18–22 nucleotides, participate in various fundamental biological processes, including cell proliferation, differentiation and apoptosis [10]. Accumulating evidence has implicated a close correlation of miRNAs with cerebral stroke. MicroRNA (miR)-152‐3p is a member of the miR‐148/152 cluster, which is involved in various cellular activities[11]. Recently, exosome miR-152-3p has benn demonstrated as a risk factor of cerebral infarction[12]. Elevated miR-152-3p expression in the hippocampus of mice following I/R was reported to be associated with the neuroprotection induced by postconditioning [13]. However, the mechanism of neuro-protection of miR-152-3p during ischemia has not yet been elucidated. According to previous investigation, miR-152-3p modulates functions of endothelial cells, such as proliferation, angiogenesis and apoptosis[14, 15].

Mitogen-activated protein kinase 3 (MAP2K3), a dual-specificity kinase in the mitogen-activated protein kinases (MAPK) family, is implicated in regulating neuro-inflammation, neuronal death and survival in brain injuries including brain ischemia [16, 17]. After searching a bioinformatics website, we determined that MAP2K3 is a possible target of miR-152-3p. However, the detailed mechanism of how MAP2K3 acts in I/R-induced BBB injury remains unclear.

In this study, we showed that miR-152-3p suppressed Bend.3 apoptosis to attenuate BBB disruption through the MAP3K2/JNK/c-Jun pathway after ischemic stroke. Our data suggested that the miR-152-3p/MAP3K2 pathway might be a potential therapeutic target for ischemic stroke.

## Materials and methods

### Animals and Groups

Adult Sprague-Dawley (SD) rats (260–280 g) (Cat# 10,395,233, Shanghai Laboratory Animal Center) were used in our study. The rats were kept in a pathogen-free room at 2nd Xiangya Hospital, Central South University (23 ± 2 ºC) with a 12-h light/dark cycle. The animals were randomized to 5 groups: the sham(sham) group, the MCAO model (MCAO) group, the agomir-NC treatment (agomir-NC + MCAO) group, and the agomir-152-3p treatment (agomir-152-3p + MCAO) group. All animal experiments complied with the ARRIVE guidelines and and should be carried out in accordance with the National Research Council’s Guide for the Care and Use of Laboratory Animals. All animal experiments were approved by the Ethics Committee of the 2nd Xiangya Hospital of Central South University.

### Intracerebroventricular(*i.c.v.*) Injection

Two hours before MCAO, agomir-152-3p/agomir-NC (5 ml of 100 mM, synthesized by GenePharma, Shanghai, China) was injected into the right lateral ventricle of rat brain (Bregma coordinates: 1.5 mm posterior, 1.8 mm lateral) at 0.2 µL/min.

### Middle Cerebral Artery Occlusion/Reperfusion (MCAO/R)

All procedures were approved by the Animal Research Committee of the 2nd Xiangya Hospital, Central South University, Hunan, China. As described previously[18], we induced tMCAO in rat models. After occlusion for 2 h the suture was withdrawn to allow reperfusion. Sham-operated rats received the same surgery without suture insertion. The animals were sacrificed 24 h after reperfusion.

### Infarct Volume Measurement and Neurological Deficits

The cerebral infarction volume was determined at 24 h of reperfusion. The brain samples were sliced into 5 coronal sections and stained with 2% 2,3,5-triphenyltetrazolium chloride (TTC, Sigma-Aldrich), as previously described [19]. Infarct size was measured using image analysis software (ImageJ, NIH, MD, USA). At 24 h after reperfusion, the sensorimotor functions of rats were assessed using the modified Garcia test as previously described[20].

### Evans Blue Dye Extravasation

Evans blue (2%, 4 ml/kg) was injected via the tail vein at 2 h before euthanasia. Rats were then transcardially perfused with 0.9% saline. The ischemic hemisphere brain tissues were homogenized in 50% ice-cold trichloroacetic acid. After centrifugation, the supernatant was collected for fluorescence spectroscopy (620 nm excitation and 680 nm emission).

### Western Blot

Rat tissues or Bend.3 cells were homogenized by sonication in RIPA buffer. Protein concentrations were determined with a BCA assay kit (QPBCA, Thermo Scientific). Protein extracts were electrophoresed by 10% SDS-PAGE. The separated protein was then electrotransferred onto PVDF membranes, shaken slowly in blocking solution (5% milk in TBST) for 90 min and incubated overnight with antibodies against occludin (1:500, Invitrogen), claudin-5 (1:1000, Abcam), MAP3K2 (1:500, Invitrogen), p-JNK1/JNK2 (1:1000, Invitrogen), JNK (1:1000, Invitrogen), p-c-Jun (1:500, Invitrogen), c-Jun (1:500, Invitrogen), active caspase-3 (1:1000, Invitrogen), caspase-3 (1:2000, Invitrogen), and β-actin (1:5000, Millipore Sigma) as a loading control. Afterwards, the membranes were washed and transferred into a buffer with appropriate secondary antibodies for 1 h at room temperature. An ECL detection kit was used to visualize the protein bands. ImageJ (LOCI, Madison, WI, USA) software was used for quantitative analysis of western blot results.

### Immunofluorescence

Brain tissues were removed after perfusion with 4% paraformaldehyde at 4 °C overnight and then immersed in 25% sucrose. Coronal Sect. (8 μm)were incubated with the following primary antibodies at 4 °C overnight: anti-occludin (1:100, Abcam), and anti-CD31 (1:200, Abcam). Sections were washed and incubated with appropriate secondary antibodies for 1 h at room temperature. In vitro cell experiments, cells were fixed with 3% glutaraldehyde, permeated with 0.3% Triton X-100 and then incubated with anti-cleaved caspase-3 (1:50, Proteintech) overnight at 4 °C. Subsequently, the cells were incubated with FITC-conjugated secondary antibody and counterstained with DAPI (1:500, Thermo Fisher Scientific).

### Cell Culture, Transfection and Oxygen-Glucose Deprivation/Reperfusion (OGD/R) Model

The well-characterized and utilized immortalized cerebral microvascular endothelial cell line (bEnd.3) (Bioleaf Biotech, Shanghai, China) was cultured in Dulbecco’s modified Eagle’s medium (DMEM; KeyGEN BioTECH, Nanjing, China) containing 15% fetal bovine serum (FBS; Gibco), and 1% penicillin/streptomycin in a humidified atmosphere of 95% air and 5% CO2 (Normoxia). After reaching 80% confluence, the cells were prepared for transfection. miR-152-3p mimics, miR-152-3p inhibitors, negative control (miR-NC or anti-miR-NC), MAK3P2 overexpressing (oe-MAK3P2) plasmid and overexpression control (oe-NC) were purchased from RiboBio (Guangzhou, China) and transfected into cells using Lipofectamine 2000 (Invitrogen, Thermo Fisher Scientific, Inc., Waltham, MA, USA) according to the manufacturer’s instructions. To develop an in vitro cerebral/ischemia model, oxygen and glucose deprivation (OGD) were induced in bend.3. The medium of bEnd.3 cells were replaced with DMEM without serum or glucose in a humidified atmosphere containing 95% nitrogen and 5% CO2 for 6 h After OGD, the cells were returned to complete medium and cultured in a normoxic incubator under 5% CO2/95% air for 6 h. The cells were randomly grouped into the control or NC (bEnd.3 without plasmids transfection or OGD/R treatment), OGD/R (OGD/R-treated bEnd.3 without transfection), OGD/R + mimics-control (OGD/R-treated bEnd.3 with transfection of mimics NC), miR-152 mimics (OGD/R-treated bEnd.3 with transfection of miR-152 mimics), OGD/R + miR-152 mimics + oe NC (OGD/R-treated bEnd.3 with cotransfection of miR-152 mimics and plasmids MAP3K2 NC), OGD/R + miR-152 mimic + MAP3K2 oe (OGD/R-treated bEnd.3 with cotransfection of miR-152 mimics and plasmids overexpressing MAP3K2).

### RT-qPCR

Total RNA was extracted using TRIzol®. Total RNA was reverse transcribed into cDNA using an RNA Reverse Transcription Kit (AB-4,366,596, Invitrogen). The expression of miR-152-3p was measured using SYBR® Premix Ex Taq™ II (Takara) (Applied Biosystems, Foster City, CA, USA). The reaction was performed as follows: 94 °C for 2 min and 40 cycles of 94 °C for 30 s, 60 °C for 20 s, and 72 °C for 20 s. U6 served as an internal reference. The data were analyzed based on the 2-ΔΔCt method[21]. According to GenBank, the primers were designed as follows (Table 1).

**Table 1:** Primer sequences

Gene	Primer sequence	
	Forward 5’-3’	Reverse 5’-3’
miR-152-3p	GCGCGGGTTCAAGACAGTAC	AGTGCAGGGTCCGAGGTATT
U6	CTCGCTTCGGCAGCACA	AACGCTTCACGAATTTGCGT

### Transendothelial Electrival Resistance (TEER) and FITC Dextran Permeation Assay

The barrier integrity of Bend.3 and FITC dextran permeation were analyzed according to the protocols detailed in our previous study[22].

### Flow Cytometry

Apoptosis of bend.3 was measured by flow cytometry using an assay kit (C1062L, Beyotime, Shanghai, China) in accordance with the manufacturer’s protocol. Briefly, Bend.3 cells were made into a cell suspension (5 × 105 cells/mL) using PBS. Then, 5 µl FITC-annexin V and 5 µl propidium iodide (PI) were added to the suspension for 10 min at room temperature without light exposure. The cells were analyzed by flow cytometry using a FACScan flow cytometry system (Becton Dickinson, San Diego, CA, USA).

### TUNEL Staining

To measure cell apoptosis, TUNEL staining (Yisheng Biotechnology, Shanghai, China) was performed according to the manufacturer’s instructions. Consecutive 1 mm2 fields in the ischemic penumbra of six randomly selected rats were photograghed with a fluorescence microscope. The final results were quantified as the number of TUNEL-positive cells/mm2 tissue.

### Luciferase Assay

The wild type of the 3′UTR of the MAP3K2 gene (containing the binding site with miR-152-3p) and mutant 3′UTR of the MAP3K2 gene were synthesized by Shanghai Beinuo Biological Technology and inserted into pmirGLO luciferase reporter vectors to generate MAP3K2 WT and MAP3K2 MUT. Luciferase assays were performed via cotransfection with pmiR-MAP3K2-WT or pmiR-MAP3K2-MAP3K2-Mut and miR-152-3p mimics using Lipofectamine 2000 (Invitrogen). After 48 h, the relative luciferase activity was measured using the dual-luciferase reporter assay system (Promega).

### Statistical Analysis

All data are shown as the mean ± standard error (SEM). Differences were evaluated using one-way ANOVA (Tukey’s multiple comparison test) or Student’s t-test (two groups). For statistical analyses, GraphPad Prism 8 (GraphPad Software) was used. p < 0.05 was considered statistically significant.

## Results

### Intracerebroventricular Injection of Agomir-Mir-152-3p Improved PostStroke Outcomes in MCAO Rats

**Fig. 1:**
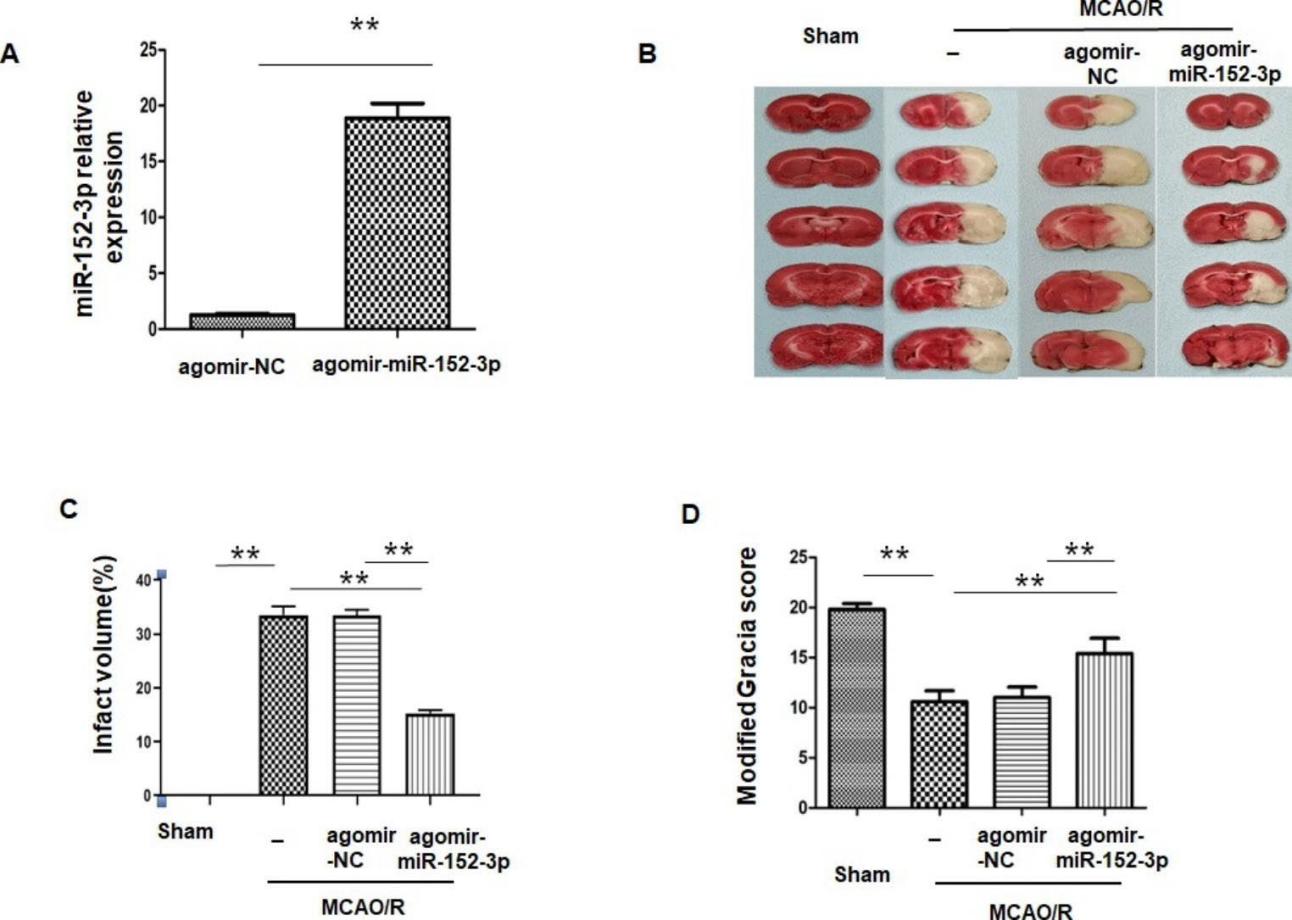
*i.c.v* injection of agomir-miR-152-3p protected against ischemic damage in MCAO rats

**Fig. 2:**
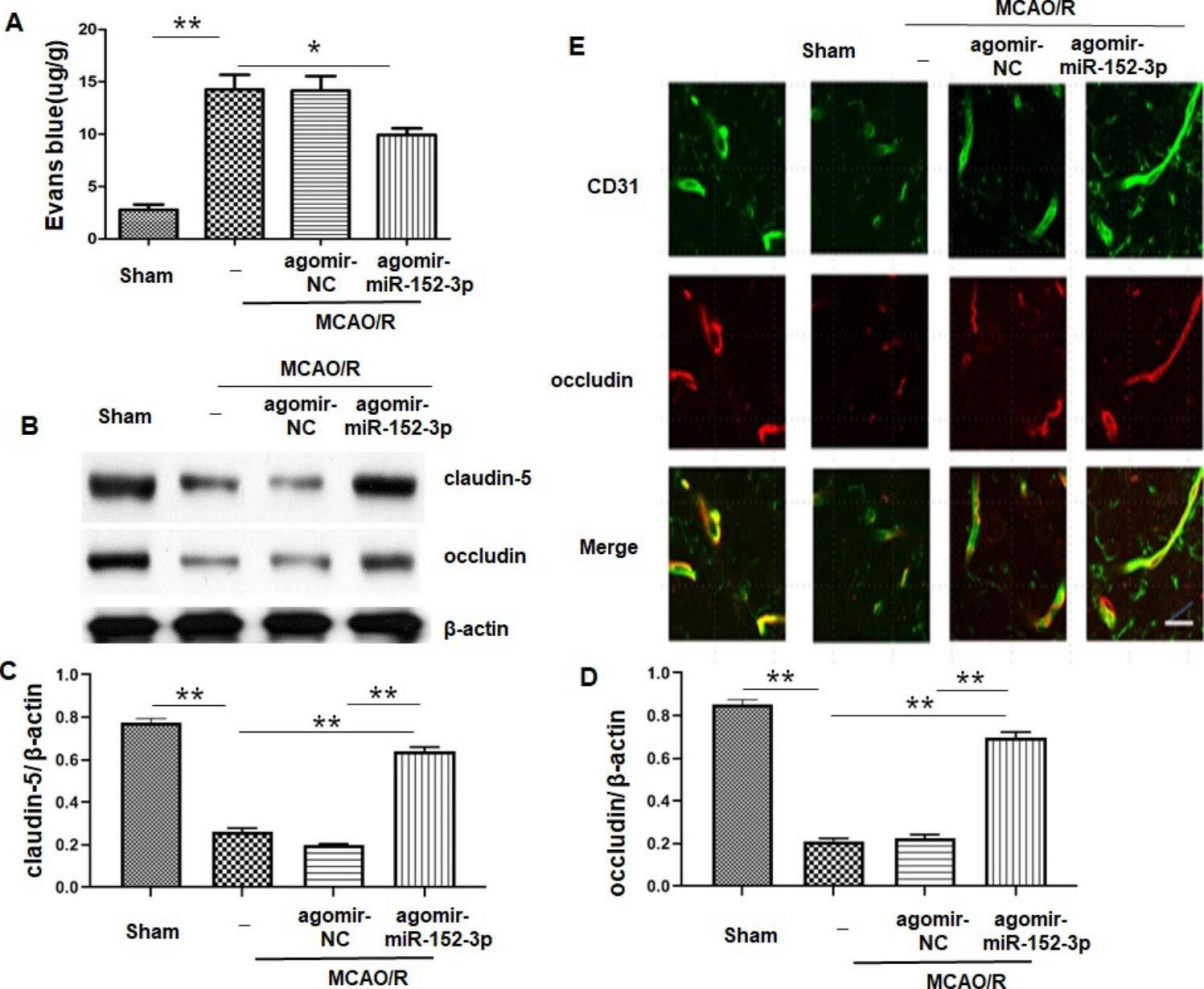
*i.c.v* injection of agomir-miR-152-3p reduced MCAO/R-induced blood–brain barrier (BBB) disruption

To explore the protective effect of miR-152-3p in vivo, the rats received an intracerebroventricular (i.c.v.) injection of agomir-miR-152-3p prior to MCAO treatment. Based on the qRT-PCR results, the miR-152-3p level was significantly higher in rats injected with agomir-miR-152-3p than in rats injected with agomir-NC (Fig. 1 A). As shown in Figs. 1B and 2 C, TTC staining of brain sections showed that the sham group exhibited no damage, while at 24 h post-MCAO, an infarcted area was obviously observed. The infarct region was significantly reduced by agomir-miR-152-3p. Moreover, after MCAO, rats exhibited significant neurological deficits compared with the sham group, while pretreatment with agomir-miR-152-3p significantly reduced neurological deficit scores compared with the MCAO group (Fig. 1D).

(A) The level of miR-152-3p in rat brain tissue was detected by RT-qPCR; (B) Representative TTC-stained brain slices in rats of different groups. (C) Quantitative results of the infarction ratio. (D) Modified Garcia scores at 24 h after MCAO. Bars represent the means ± SEM. n = 5 per group. **P < 0.01, compared with the indicated group.

### *I.C.V* Injection of Agomir-miR-152-3p Blunted Bbb Damage After MCAO/R

Evans blue (EB) extravasation was used to evaluate BBB disruption. We observed that EB leakage was increased in the MCAO + agomir-NC group compared to the sham group, while agomir-miR-152-3p pretreatment remarkably reduced MCAO-induced EB extravasation (Fig. 2 A). Tight junction proteins (TJPs), such as occludin and claudin-5, are strongly related to BBB stability[23]. Hence, the expression levels of occludin and claudin-5, the major TJPs in the membrane of BMECs, were also assessed by western blot in the present study. The levels of occludin and claudin-5 were significantly decreased after MCAO compared with the sham group, and this effect was significantly ameliorated by miR-152-3p (Fig. 2B–D). Moreover, we conducted immunofluorescence, labeling claudin-5 and CD31, a marker of endothelial cells to observe tight junction distribution in situ. Results indicated that occludin/CD31 double immunofluorescence staining revealed that MCAO induced lower intensities of occludin and junctional discontinuity than sham. The degradation of TJPs was reversed by miR-152-3p (Fig. 2E).

A. Quantitative analysis of Evans blue (EB) leakage in ischemic brains at 72 h after MCAO. B-D.

Representative western blot bands and quantitative analysis of Claudin-5 and occludin. E. Representative immunofluorescent images of the colocalization of occludin in CD31 + endothelial cells. n = 5 per group. Bars represent the means ± SEM. *P < 0.05, compared with the indicated group; **P < 0.01, compared with the indicated group.

### 3.3 miR-152-3p Alleviated BBB Disruption After OGD/R

**Fig. 3:**
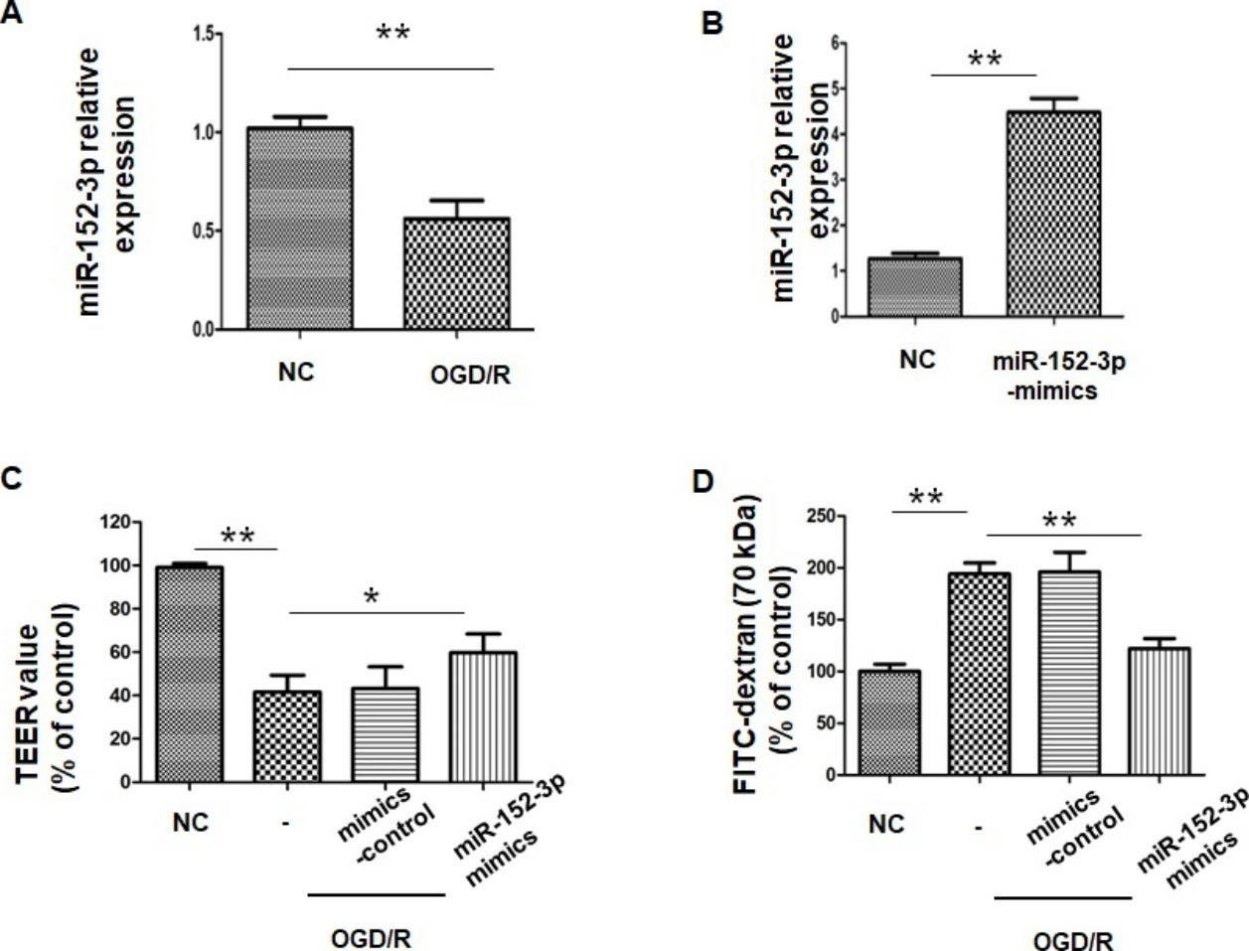
Overexpression of miR-152-3p protected the OGD/R-induced disruption of BBB integrity in vitro

A monolayer of Bend.3 grown in the Transwell insert was employed as an in vitro blood-brain barrier model. To examine the neuroprotective effect of miR-152-3p during OGD/R, we first investigated miR-152-3p expression in bEnd.3 cells under OGD/R conditions. RT-qPCR results showed that miR-152-3p expression was markedly downregulated following OGD/R (Fig. 3 A), indicating that miR-152-3p may be associated with BBB pathology after OGD/R. To investigate the effect of miR-152-3p on barrier function after OGD/R injury, a gain-of-function experiment was performed by transfecting miR-152-3p mimics into bEnd.3 cells. miR-152-3p expression was upregulated by miR-152-3p mimics, as confirmed by RT-qPCR (Fig. 3B). We observed that OGD/R caused a significant increase in paracellular permeability to FITC- (4 kDa) dextrans across bend.3 monolayers compared to the control group, while miR-152-3p overexpression reduced the permeability compared to the OGD/R group (Fig. 3 C). In line with the paracellular permeability results, the TEER value in OGD/R-exposed endothelial monolayers was significantly decreased compared with that in the control group. However, miR-152-3p protected endothelial monolayers from the OGD-induced decrease in TEER (Fig. 3D).

A. The levels of miR-152-3p in Bend.3 were analyzed via qRT-PCR. B. The levels of miR-152-3p after.

transfection were analyzed via qRT-PCR. C. TEER assay of monocultures in different groups. D. The permeability of fluorescein isothiocyanate (FITC)-dextran across bEnd.3 cells. n = 5 per group. Bars represent the means ± SEM. *P < 0.05, compared with the indicated group; **P < 0.01, compared with the indicated group.

### miR-152-3p Decreased Bend.3 Apoptosis After OGD/R

**Fig. 4:**
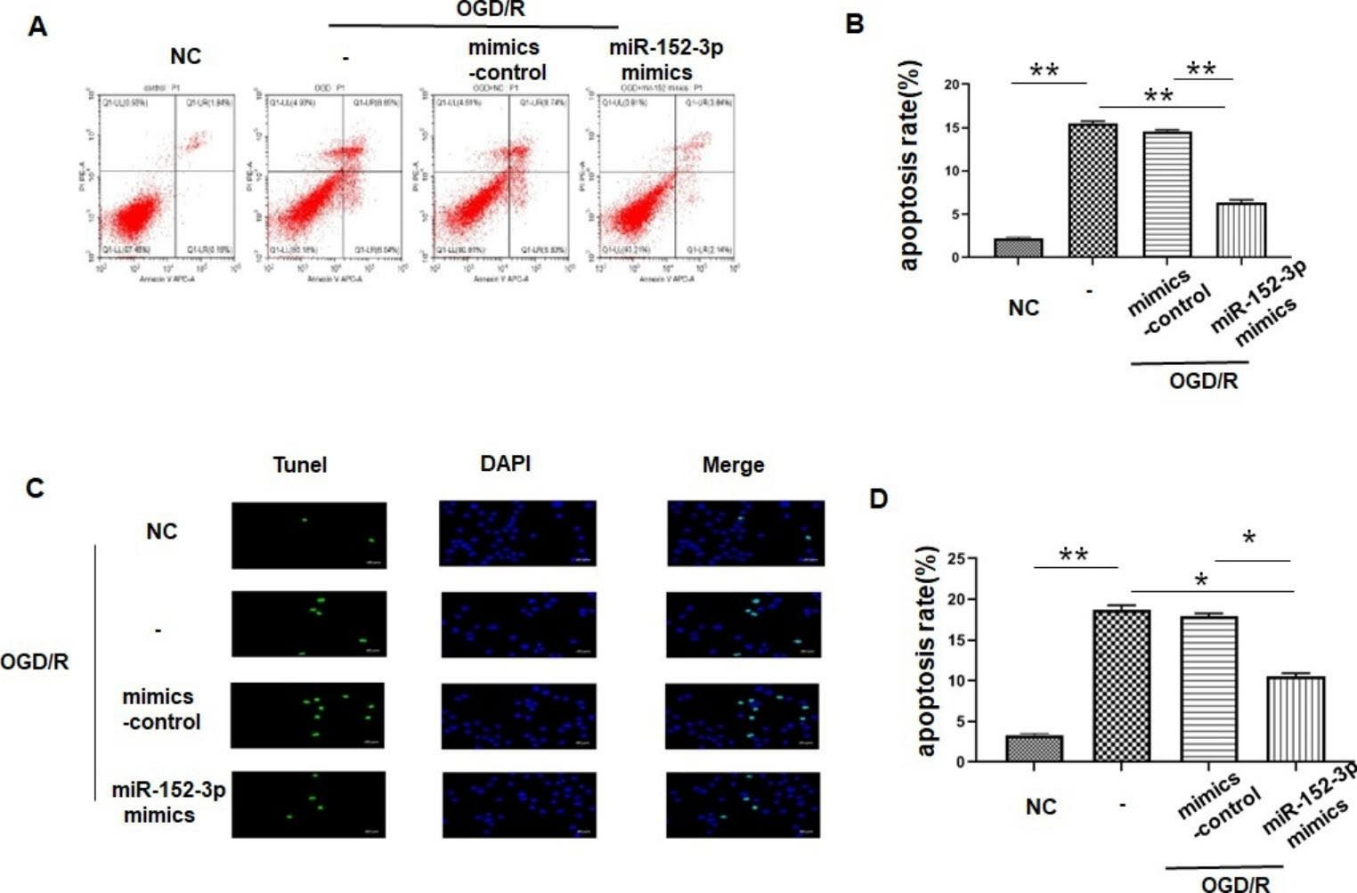
A. Apoptosis was assessed by flow cytometry. B. The percentage of apoptotic cells. C-D. Apoptosis rate of Bend.3 evaluated by the TUNEL assay. Bars represent the means ± SEM. n = 3 per group. *P < 0.05, compared with the indicated group; **P < 0.01, compared with the indicated group

Previous studies indicated that apoptosis of BMECs during ischemic injury contributes to compromised formation of TJs and loss of microvascular integrity[24, 25]. The results of flow cytometric analysis showedthat the number of apoptotic cells was significantly increased after OGD/R compared with that in the control group (Fig. 4 A-B). Overexpression of miR-152-3p partially reversed the apoptosis of bend.3 in the OGD/R group. Furthermore, a large number of TUNEL-positive cells were observed in bend.3 in the OGD/R group, while the number of TUNEL-positive cells was remarkably reduced in the miR-152-3p group compared with the OGD/R group (Fig. 4 C-D).

### MAP3K2 is a miR-152-3p Target Gene

**Fig. 5:**
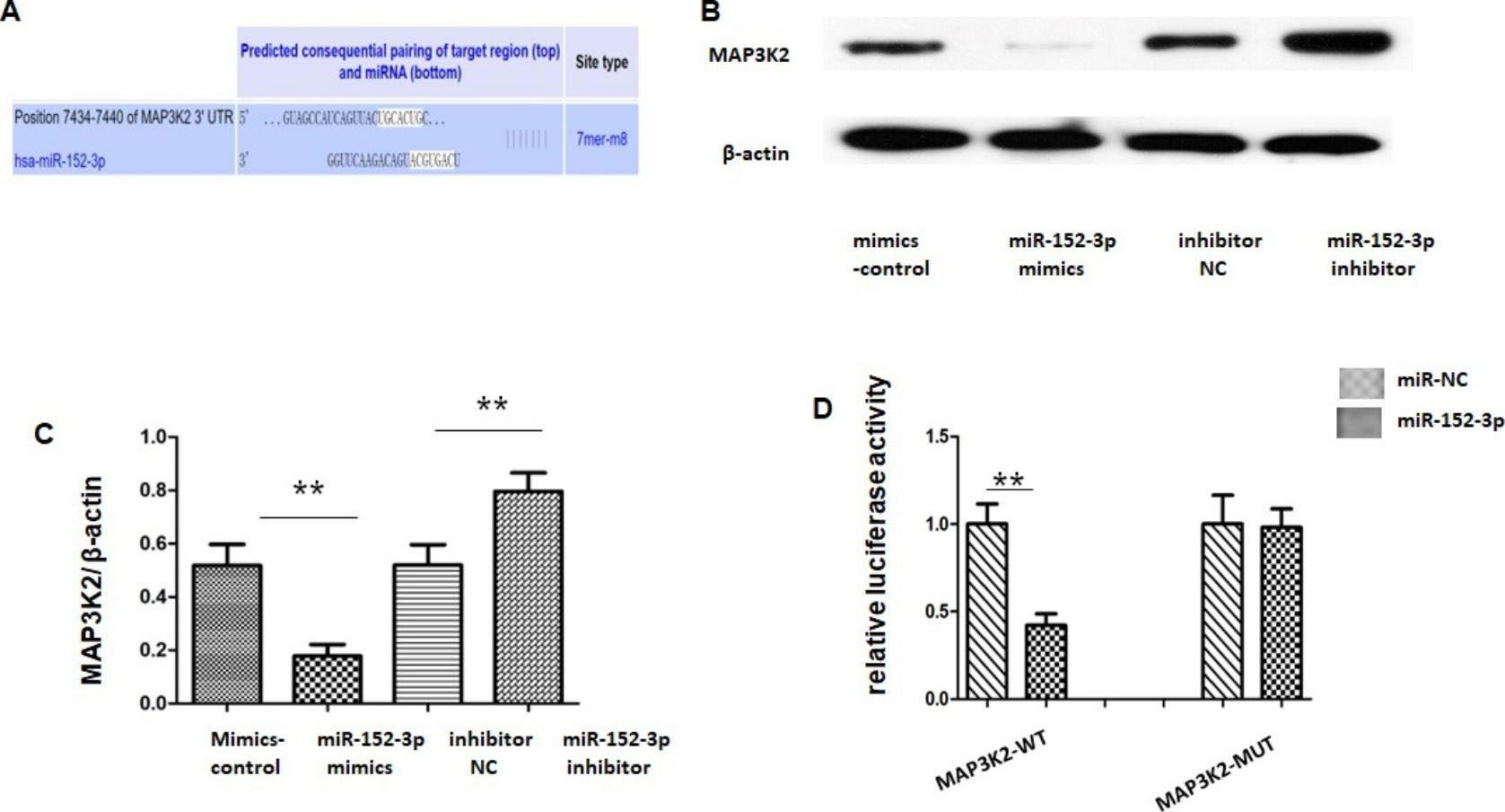
**miR-152-3p targeted MAP3K2.**

To uncover the mechanism underlying the protective effect of miR-152-3p during OGD/R, downstream targets of miR-152-3p were predicted by the online software TargetScan (www.targetscan.org). Among these predicted targets, MAP3K2 attracted our interest as an important regulator of apoptosis. The 3ʹ-UTR of MAP3K2 mRNA possesses a conserved binding site for miR-152-3p (Fig. 5 A). We further investigated the relationship between miR-152-3p and MAP3K2 in bend.3. The protein level of MAP3K2 was decreased after overexpression of miR152-3p with miR152-3p mimics and increased after suppression of miR152-3p with miR152-3p inhibitor (Fig. 5B-C). To further explore whether miR-152-3p could directly bind to the 3′-UTR region of MAP3K2 mRNA, a luciferase assay was performed inHEK293 cells, in which the 3′-UTR fragment of MAP3K2 mRNA was used to construct the psiCHECK vector. As expected, miR-152-3p mimics significantly decreased the luciferase activity of pMAP3K2-WT in HEK293 cells, without any effect on the luciferase activity of pMAP3K2-MUT (Fig. 5D).

(A) Bioinformatics analysis of the potential complementary site of miR-152-3p on MAP3K2 (B) Representative western blot bands of MAP2K4 protein levels. (C) Analysis of relative protein levels of MAP3K2. (D) The luciferase reporter assay revealed luciferase activity of Wt/Mut pmirGLO-MAP3K2-3′UTR after upregulating miR-152-3p. Bars represent the means ± SEM. n = 3 per group. **P < 0.01, compared with the indicated group.

### miR-152-3p Inhibited Apoptosis of Bend.3 and Improved the Integrity of the BBB by Negatively Regulating MAP3K2

**Fig. 6:**
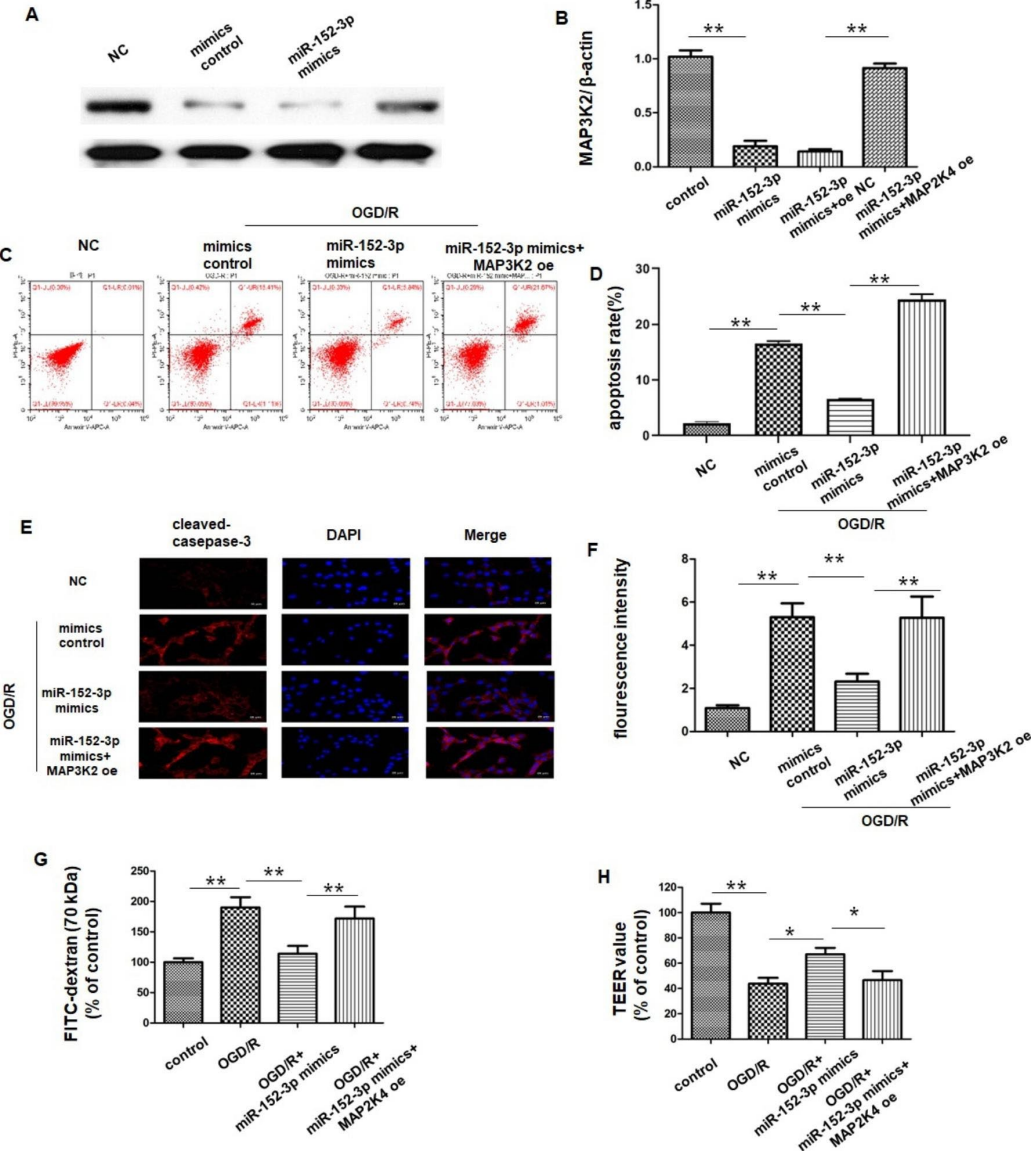
miR-152-3p inhibited Bend.3 apoptosis by targeting MAP3K2

The present study also aimed to determine whether MAP3K2 was involved in the protective action of miR-152-3p. We treated bend.3 cells with miR-152-3p mimics, miR-152-3p mimics + oe-NC or miR-152-3p mimics + oe-MAK3P2 before OGD/R. Western blot analysis showed that the expression of MAP3K2 was downregulated by miR-152-3p mimic transfection. However, this effect was reversed by cotransfection with miR-152-3p mimics and oe-MAP3K2 (Fig. 6 A-B). The results of flow cytometry indicated that transfection with oe-MAP3K2 partially reversed the anti-apoptotic effect of miR-152-3p after OGD/R (Fig. 6 C-D). Similarly, the fluorescence intensity of cleaved caspase-3 demonstrated that caspase-3 activation was significantly decreased by miR-152-3p mimics compared with the OGD/R group, which was reversed by cotransfection with miR-152-3p mimics and oe-MAP3K2 (6E-F).

As previously demonstrated, OGD/R noticeably increased the permeability of FITC dextran across BMEC monolayers, while miR-152-3p mimic pretreatment significantly offset this effect. Compared with the OGD/R-treated miR-152-3p mimics group, cotransfection with miR-152-3p mimics and oe-MAP3K2 noticeably increased the permeability of FITC dextran across Bend.3 monolayers (Fig. 6G). Similarly, miR-152-3p inhibited the OGD/R-induced TEER decrease, while overexpression of MAP3K2 partially antagonized the effect of miR-152-3p (Fig. 6 H).

A-B. Representative western blot bands and quantitative analysis of MAP3K2 protein expression in oe-MAP3K2- or oe-NC-transfected cells. C-D. Apoptosis of Bend. 3 after OGD/R in different groups. E. Representative immunofluorescent images of cleaved caspase-3 and DAPI immunofluorescent staining. F. Quantification of cleaved caspase-3-positive cells. G. TEER assay of moncultures in different groups. H. The permeability of fluorescein isothiocyanate (FITC)-dextran across bEnd.3 cells. n = 5 per group. Bars represent the means ± SEM. n = 3 per group. *P < 0.05, compared with the indicated group; **P < 0.01, compared with the indicated group.

### miR-152-3p Modulated the JNK/c-Jun Pathway by Targeting MAP3K2 After OGD/R

**Fig. 7:**
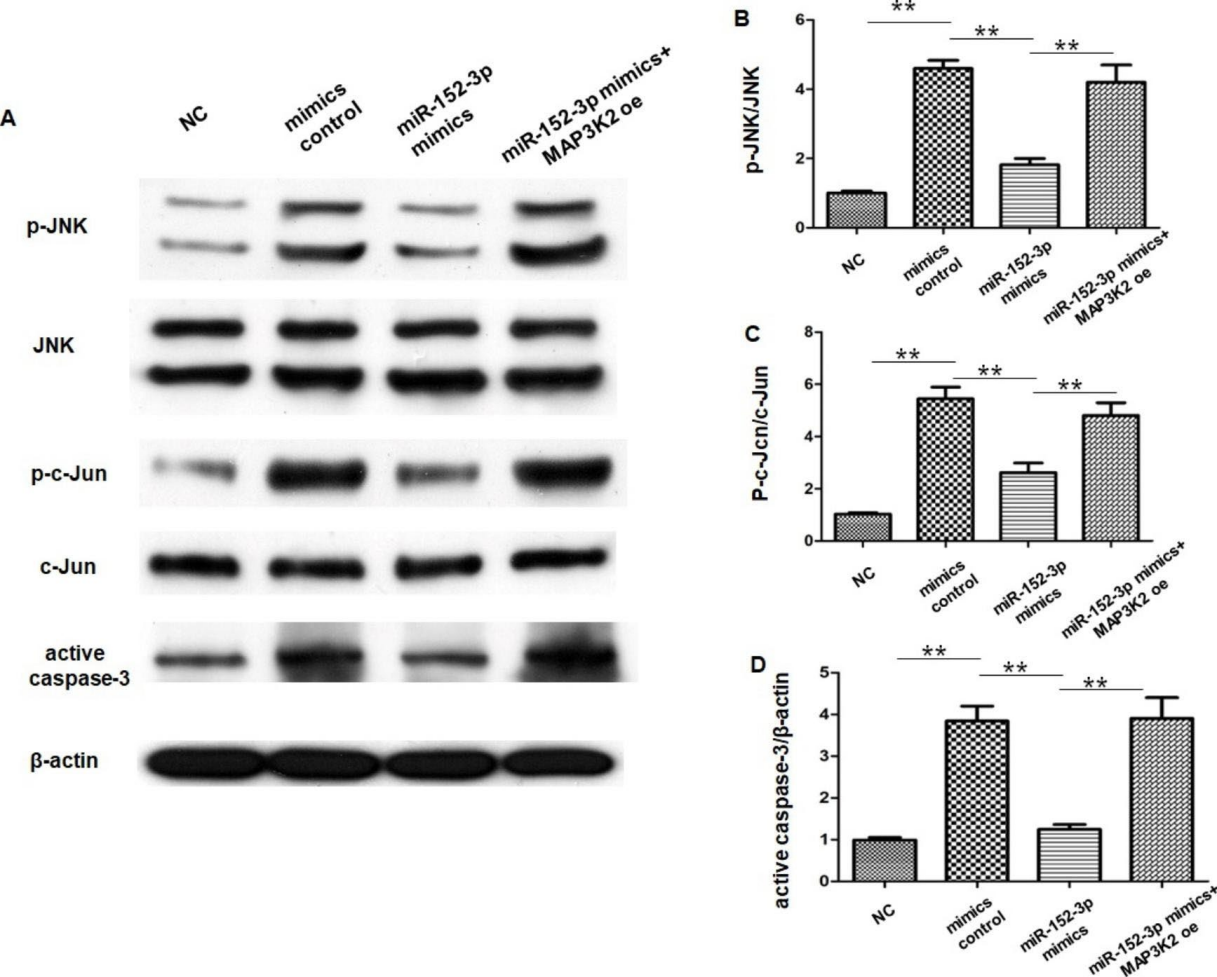
miR-152-3p inhibited activation of the JNK/c-Jun pathway by targeting MAP3K2

MAP3K2 has been found to be an activator of JNK/c-Jun pathway, which has been reported to promote apoptosis in endothelial cells. We speculated that MAP3K2 may regulate the JNK/c-Jun pathway to attenuate Bend.3 apoptosis after OGD/R. As illustrated in Fig. 8 A-C, compared with the NC group, OGD/R significantly increased the phosphorylation of JNK and p-c-Jun and upregulated active caspase-3; while overexpression of miR-152-3p significantly inhibited the p-JNK/JNK and p‐c‐Jun/c‐Jun ratio and decreased the active caspase-3 compared with the OGD/R group. In cells cotransfected with miR-152-3p mimics and oe-MAP3K2, the levels of p-JNK/JNK, p-c-Jun/Jun and activated caspase-3 were increased again (Fig. 7 A-D). These results suggested that miR-152-3p modulated the JNK/c-Jun signaling pathway after OGD/R, and that MAP3K2 mediated this effect.

(A) Expression of p-JNK, JNK, p-c-Jun, c-Jun and active caspase-3 detected by western blot. (B) Quantitative analysis of p-JNK/JNK. (C) Quantitative analysis of p-c-Jun/c-Jun. (D) Quantitative analysis of active caspase-3. Bars represent the means ± SEM. n = 3 per group. **P < 0.01, compared with the indicated group.

## Discussion

Recently, the beneficial effect of miR-152-3p during brain ischemia has been demonstrated, but its potential impact on ischemia-induced BBB breakdown has remained elusive. BBB disruption following acute ischemic stroke is the primary cause of brain edema and hemorrhagic transformation, which contributes to worse stroke outcome[26, 27]. Studies have increasingly suggested that BBB protection is important for ischemic stroke therapy[28]. We found that miR-152-3p played a protective role against MCAO-induced BBB collapse in rats and OGD-stimulated endothelial barrier damage. Mechanistically, miR-152-3p inhibited bend.3 apoptosis by targeting MAP3K2.

In previous investigations, the expression of serum exosomal miR-152-3p in patients withacute ischemic stroke was significantly lower than that in healthy controls[12]. In addition, miR-152-3p was found to be downregulated in the hippocampus of I/R[13]. These data indicate that miR-152-3p may be involved in the regulation of ischemic injury. As expected, preischemic intracerebroventricular injection of agomir-miR-152-3p improved stroke outcomes by reducing infarct volume and improving neurological functions in our study. Importantly, miR-152-3p protected against BBB destruction following MCAO. evidenced by reduced EB extravasation from the BBB. BBB failure can be characterized by reduced levels of TJPs, such as claudin-5 and occludin, after ischemia[6, 29]. In the current study, we also demonstrated that miR-152-3p attenuated MCAO-induced TJP degradation and improved TJP integrity in occludin immunofluorescence localization.

BMECs play an important role in BBB function by establishing a highly selective barrier[30]. We discovered that miR-152-3p expression was downregulated in OGD/R‐treated Bend.3 cells, implying that miR-152-3p may play a role in the pathological process of the BBB. Previous studies have shown that ischemic stroke leads to Bend.3 apoptosis associated with BBB disruption[31–33]. Hence, protecting endothelial cells can maintain the integrity of the BBB.

miR-152-3p role in apoptosis is still controversial. A number of evidence highlight the importance of miR-152-3p as a tumor suppressor. miR-152 promoted apoptosis of ovarian cancer cells through repression of ERBB3 expression[34]. miR-152 overexpression caused growth inhibition and increased cell apoptosis in TamR MCF-7 cells via downregulating ALCAM[35]. miR-152-3p decreased the survival rate and increased the apoptosis rate of MCF-7/TAX cells to paclitaxel via negatively regulating EPAS1[36]. Moreover, in sepsis-induced acute kidney injury, miR-152-3p was reported to aggravate apoptosis and inflammation by targeting ERRFI1[37].However, recent evidence shows that miR-152 has an antiapoptotic action in different models. miR-152 attenuated lncRNA PVT1-induced apoptosis via c-MET/PI3K/AKT pathway in osteosarcoma cell[38]. miR-152-3p regulates TCF-4 pathway in OA rats to weaken chondrocyte apoptosis[39]. miR-152-3p overexpression suppressed angiotensin II-induced[40] and hypoxia-induced cardiomyocyte apoptosis in vitro[41]. A recent study revealed that miR-152-3p overexpression protected neurons from OGD/R-induced apoptosis[42]. In our study, miR-152-3p elicited anti-apoptotic effect on Bend.3 under OGD/R condition, which was confirmed by flow cytometry and TUNEL staining, which was not consistent All these observations identify that the function of miR-152-3p in apoptosis is complex, being proapoptotic or antiapoptotic, probably depending on cell type, context and the apoptotic cues.

Since miRNAs do not code for proteins, the protective effect of miR-152-3p on cerebral I/R injury is assumed to be exerted via the repression of specific proteins. In the present study, we provided evidence that miR-152-3p could target MAP3K2 to exert its protective action by the following experiments: (1) With the assistance of bioinformatics tools (http://www.targetscan.org/vert_72/), MAP3K2 was identified as the target of miR-152‐3p; (2) suppression of miR-152-3p promoted MAP3K2 expression, whereas overexpression of miR-152-3p downregulated MAP3K2 expression in bend.3; (3) miR-152-3p mimics significantly regulated the luciferase activity of MAP3K2-WT but not MAP3K2-MUT; (4) Rescue experiments showed that restoration of MAP3K2 expression partially attenuated the anti-apoptosis impact induced by miR-152-3p overexpression.

MAP3K2 is an upstream kinase in the mitogen-activated protein kinase (MAPK) signaling pathway that preferentially regulates the JNK and ERK5 pathways. Multiple lines of evidence have linked MAP3K2 to apoptosis[43, 44]. Notably, MAK3P2 may induce distinct effects on apoptosis. The MAP2K5/ERK5 pathway improves EC viability and confers resistance to apoptosis [45, 46]; conversely, JNK MAPKs play a proapoptotic role in ECs under most conditions [47]. Targeted disruption of the JNK pathway has been reported to be involved in anti-apoptotic mechanisms, which reduce cerebral I/R injury[20, 48]. c-Jun is the major downstream substrate of the JNK pathway. Under the action of JNK, phosphorylated c-Jun further promotes the expression of a variety of pro-apoptotic proteins[49]. In this study, we found that miR-152-3p positively modulated the phosphorylation state of JNK and c-Jun and decreased the expression of active caspase-3. This phenomenon was reversed by MAP3K2 overexpression. Therefore, the effects of miR-152-3p on endothelial apoptosis during OGD/R may be induced by MAP3K2 suppression through the JNK/c-Jun pathway.

This is our first attempt to reveal the role of miR-152-3p during cerebral I/R and the relevant pathway in BBB protection. Despite these promising results in vitro, the relationship of miR-152-3p and MAP3K2 was not investigated in vivo animal experiments, which will be included in our future studies. In addition, the expression detection in blood samples might provide more information in clinic.

## Conclusion

In summary, miR-152-3p attenuated BBB damage under brain ischemia. Mechanistically, miR-152-3p attenuated OGD/R-induced endothelial apoptosis by regulating the MAP3K2-mediated JNK/c-Jun pathway. The anti-apoptotic effect of miR-152-3p might serve as a potential therapeutic target for cerebral I/R injury.

## Data Availability

The data used to support the findings of this study are included within the article.
