# Correction to: MicroRNA152-3p Protects Against Ischemia/Reperfusion-Induced Bbb Destruction Possibly Targeting the MAP3K2/JNK/c-Jun Pathway

**DOI:** 10.1007/s11064-023-03919-7

**Published:** 2023-04-15

**Authors:** Fei Li, Fangfang Zhou, Binbin Yang

**Affiliations:** 1 Department of Neurology, Changzheng Hospital, Naval Medical University, Shanghai, China; 2 Department of Neurology, 2nd Xiangya Hospital, Central South University, Changsha, Hunan China

**Correction to: Neurochemical Research** 10.1007/s11064-022-03828-1

In the original article, under section “Results” there was an error in Fig. 2E. The correct version of Fig. 2E is given below.

**Fig. 2:**
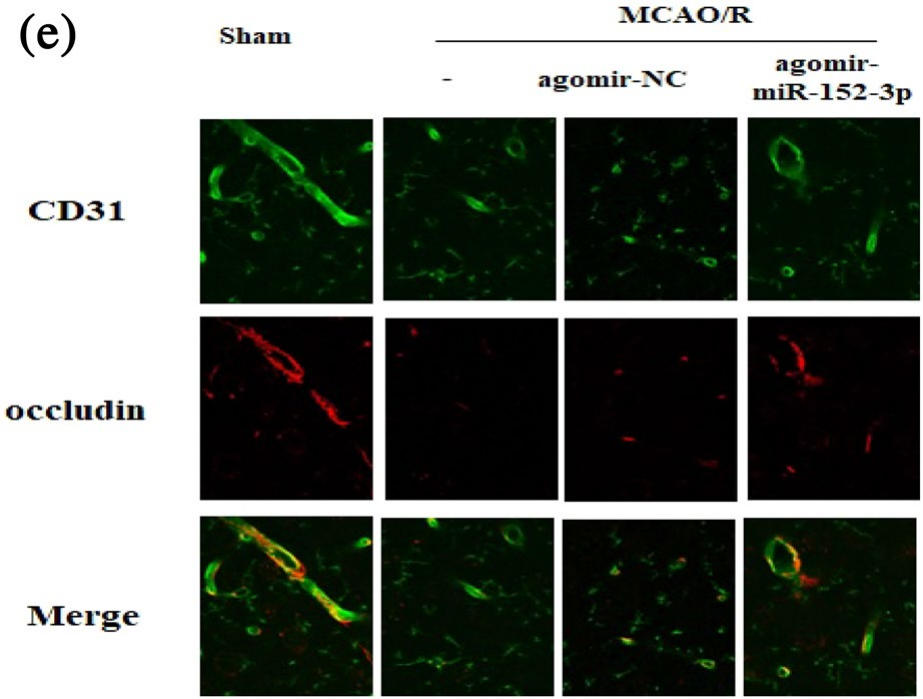
*i.c.v* injection of agomir-miR-152-3p reduced MCAO/R-induced blood–brain barrier (BBB) disruption

The experiments were run in triplicate, and the data are shown as followings.

This has been corrected by publishing this correction article.

